# Characterization and development of EST-SSR markers in sweet potato (*Ipomoea batatas* (L.) Lam)

**DOI:** 10.1007/s13205-016-0565-9

**Published:** 2016-11-12

**Authors:** Jin-Hee Kim, Jun-Hoi Kim, Won-Sam Jo, Jeong-Gwan Ham, Il Kyung Chung, Kyung-Min Kim

**Affiliations:** 1 School of Applied Biosciences, College of Agriculture and Life Sciences, Kyungpook National University, Daegu, 41566 South Korea; 2 Department of Biotechnology, Catholic University of Daegu, Gyeongsan-Si, Gyeongbuk 38430 South Korea

**Keywords:** EST-SSR marker, Hexaploid, Sweet potato, Polymorphic

## Abstract

In this study, a cDNA library was constructed from the total RNA of sweet potato leaves. A total of 789 copies of the cDNA were cloned in *Escherichia coli* by employing the pGEM-T Easy vector. Sequencing was carried out by Solgent Co. (Korea). As many as 579 expressed sequence tag–simple sequence repeat (EST-SSR) markers were designed (73.38%) from the known cDNA nucleotide base sequences. The lengths of the developed EST-SSR markers ranged from 100 to 499 bp (average length 238 bp). Their motif sequence types were varied, with most being dinucleotides and pentanucleotides, and the most commonly found motifs were CAGAAT (29.0%) and TCT (2.8%). Based on these SSR-containing sequences, 619 pairs of high-quality SSR primers were designed using WebSat and Primer3web. The total number of primers designed was 144. Polymorphism was evident in 82 EST-SSR markers among 20 Korean sweet potato cultivars tested and in 90 EST-SSR markers in the two parents of a mapping population, Yeseumi and Annobeny. In this study, the hexaploid sweet potato (2*n* = 6*x* = 90) EST-SSR markers were developed in the absence of full-sequence data. Moreover, by acting as a molecular tag for particular traits, the EST-SSR marker can also simultaneously identify information about the corresponding gene. These EST-SSR markers will allow the molecular analysis of sweet potato to be done more efficiently. Thus, we can develop high-quality sweet potato while overcoming the challenges from climate change and other unfavorable conditions.

## Introduction

Sweet potato [*Ipomoea batatas* (L.) Lam.] is a hexaploid (2*n* = 6*x* = 90) plant that belongs to the family Convolvulaceae (Ting and Kehr 1953; Martin 1970). The tuberous root of sweet potato, which is involved in carbohydrate storage and vegetative propagation, is a unique organ that has value in biological research of organogenesis and evolution as well as importance in agriculture. In the future, more variations of sweet potato will be needed for its breeding, because despite its importance, this hexaploid crop is difficult to breed owing to the complexity of its genetics and the lack of genomic resources (Kriegner et al. 2003; Cervantes-Flores et al. 2008). Traditionally, phenotypic markers have been used to provide descriptors for identifying sweet potato cultivars (Huamán and Zhang 1997), but they are unreliable owing to their paucity and vulnerability to environmental influence. Molecular markers have great potential to speed up the process of developing improved cultivars; however, a little effort has been devoted to the development and application of molecular marker technology for the genetic improvement of sweet potato (Kriegner et al. 2003). Genetic markers offer a number of applications for sweet potato genetic improvement. The randomly amplified polymorphic DNA (RAPD) technique developed in 1990 is a powerful molecular marker technique in genetics and plant breeding (Welsh and McClelland 1990; Williams et al. 1990). RAPD markers have been used on sweet potato to study genetic segregation and linkage (Thompson et al. 1997), construct DNA fingerprints (Connolly et al. 1994), and identify a gene for root-knot nematode resistance (Ukoskit et al. 1997). The amplified fragment length polymorphism (AFLP) technique developed in 1995 (Vos et al. 1995) is based on the selective PCR amplification of small restriction fragments (80–400 bp) from a total digest of genomic DNA. In sweet potato, AFLP markers have been used to study genetic linkage maps (Kriegner et al. 2003; Cervantes-Flores et al. 2008) and to assess genetic diversity (Zhang et al.^ 2000^). Expressed sequence tags (ESTs) can be used as a cost-effective and valuable source for the development of molecular markers, such as single nucleotide polymorphisms (SNPs) and simple sequence repeats (SSRs). DNA SSRs are widely distributed in both noncoding and transcribed sequences, and are commonly known as genomic-SSRs and EST-SSRs (Morgante et al. 2002). SSRs are useful for many applications in plant genetics and breeding, such as for high-density linkage map construction, genetic diversity analysis, cultivar identification, and marker-assisted selection. However, it is still expensive, labor intensive, and time-consuming to develop genomic SSR markers. In contrast, EST-SSRs can be rapidly developed from an EST database at lower cost. Moreover, EST-SSRs can also lead to direct gene tagging for quantitative trait locus mapping of agronomically important traits and increase the efficiency of marker-assisted selection (Gupta and Rustgi 2004). In addition, EST-SSRs show a higher level of transferability to closely related species than do genomic SSR markers (Scott et al. 2000; Eujayl et al. 2004; Zhang et al. 2005; Saha et al. 2006) and can serve as anchor markers for comparative mapping and evolutionary studies (Varshney et al. 2005a, b). Breeding practices for improving the quality of sweet potato is ongoing worldwide, and the recent molecular markers developed for sweet potato have demonstrated good potential to be used in genetic selection (Wang et al. 2010).

## Materials and methods

### Plant materials

In this study, the 20 Korean sweet potato cultivars and two parents of a mapping population, Yeseumi and Annobeny, were provided by the Rural Development Administration (Jeollabuk, Korea) (Table 1).

**Table 1:** Sweet potato cultivars used for EST-SSR marker validation and evaluation

Cultivar name	Origin	Description
Yeseumi	Korea	Improved variety, mapping parent
Annobeny	Japan	Introduced variety, mapping parent
Yulmi	Korea	Improved variety
Jeonmi	Korea	Improved variety
Gogeonmi	Korea	Improved variety
Jungmi	Korea	Improved variety
Sincheonmi	Korea	Improved variety
Geonhwangmi	Korea	Improved variety
Yeonmi	Korea	Improved variety
Geonmi	Korea	Improved variety
Yeonjami	Korea	Improved variety
Sinjami	Korea	Improved variety
Sinyulmi	Korea	Improved variety
Geonpungmi	Korea	Improved variety
Helseumi	Korea	Improved variety
Hayanmi	Korea	Improved variety
Jinhongmi	Korea	Improved variety
Juhwangmi	Korea	Improved variety
Dahomi	Korea	Improved variety
Simgeonmi	Korea	Improved variety
Yeonhwangmi	Korea	Improved variety

### DNA and RNA extraction

Total RNA was extracted from leaf tissue of sweet potato using the RNeasy Mini Kit (Qiagen, Hilden, Germany) according to the manufacturer’s instructions. In brief, RLT buffer (Qiagen) containing β-mercaptoethanol was thoroughly mixed with the sample. The mixture was then transferred to a QIAshredder spin column and centrifuged for 5 min. The supernatant was transferred to a new 1.5 mL tube and an equal volume of 70% ethyl alcohol was added. The mixture was immediately applied to an RNeasy spin column that was then centrifuged for 1 min at 13,000 rpm. The flow-through was discarded and the column was washed by adding 500 μL of RPE buffer (Qiagen) followed by centrifugation for 1 min at 13,000 rpm. The flow-through was again discarded and the washing process was repeated. Finally, the RNA was suspended in 30 μL of RNase-free water. The concentration of genomic DNA was checked using a Nano Drop 2000 spectrophotometer. The genomic DNA was extracted from leaf tissue of sweet potato using a modified cetyltrimethyl ammonium bromide (CTAB) method. Samples of 20–100 mg of leaves were placed, respectively, in a 2 mL tube, containing a tungsten ball and frozen liquid nitrogen, for 5 min. The samples were then ground into powder using a TissueLyser apparatus (Qiagen) at 20 vibrations per second for 30 s. Next, 700 μL of 2× CTAB buffer (2% CTAB, 0.1 M Tris, pH 8.0, 1.4 M NaCl, 1% polyvinylpyrrolidone) was added to the tubes. The samples were then vortexed, after which the tubes were incubated in a water bath at 65 °C for 20 min. After removal from the water bath, 700 μL of phenol:chloroform:isoamyl alcohol PCI (25:24:1) was added to the samples and the tubes were shaken for 20 min at room temperature before centrifugation. Next, 500 μL of the supernatant was removed to a new 1.5 mL tube and 350 μL of isopropanol was added. The tubes were then shaken for 5 min, followed by freezing at −72 °C for 2 h. Subsequently, the samples were melted slowly and centrifuged at 13,000 rpm for 10 min. After discarding the supernatant, the pellet was dried at room temperature after washing two times in 70% ethanol. Finally, 20 μL of distilled water was added to each tube and the concentration of genomic DNA was checked using a Nano Drop 2000 spectrophotometer.

### Construction of the cDNA library

The cDNA library was synthesized using a cDNA synthesis kit (TaKaRa, Shiga, Japan). In brief, 2000 ng of template RNA was added to a mixture composed of 4 μL of 5× 1st strand synthesis buffer, 1 μL of dNTP mixture (10 mM), 1 μL of RNase inhibitor (20 U/μL), 2 μL of oligo(dT)18 primer (1 μg/μL), and 1 μL of M-MLV reverse transcriptase, in a total volume of 20 μL. The mixture was then subjected to the following first-strand cDNA reaction conditions: room temperature for 10 min, 42 °C for 1 h, and 80 °C for 5 min. Next, the mixture was mixed with 30 μL of 5× 2nd strand synthesis buffer and 3 μL of dNTP mixture (10 mM), after which the volume was adjusted to 89 μL with nuclease-free water. Then, 2 μL of *Escherichia coli* DNA polymerase I (20 U/μL) and 2 μL of *E. coli* RNase H/*E. coli* DNA ligase mixture were added, and the mixture was incubated at 16 °C for 2 h and 70 °C for 10 min. Subsequently, 4 μL of T4 DNA ligase (1 U/μL) was added to the solution and incubation was carried out at 37 °C for 10 min. The reaction was stopped by the addition of 12 μL of stop solution (0.2 M ethylenediaminetetraacetic acid and 2 mg/mL glycogen, pH 8.0). To purify the cDNA, an equal volume of phenol:chloroform:isoamyl alcohol (25:24:1) was then added to the solution, after which the mixture was vortex-mixed and then centrifuged at 13,000 rpm for 10 min. The upper layer was transferred to a new 1.5 mL tube and an equal volume of chloroform:isoamyl alcohol (24:1) was added. The tube was stirred for 1 min and then centrifuged at 13,000 rpm for 10 min. The upper layer was transferred to a new 1.5 mL tube and an equal volume of 4 M ammonium acetate was added, followed by an equal volume of isopropanol. Following incubation at −20 °C for 30 min, the mixture was centrifuged at 13,000 rpm for 10 min. The supernatant was subsequently removed, 1 mL of ethyl alcohol (70%) was added to the pellet, and the suspension was centrifuged at 13,000 rpm for 5 min. The cDNA was finally suspended in nuclease-free water. The cDNA was A-tailed in a total 10 μL reaction volume by adding 1 μL of 10× buffer, 0.6 μL of MgCl2 (25 mM), 0.4 μL of dATP (5 mM/μL), and 1 μL of Taq polymerase (5 U/μL). The mixture was incubated at 70 °C for 30 min and then purified using ethyl alcohol.

### cDNA Library transformation to the vector

The cDNA library was ligated to the pGEM-T Easy vector (Promega, Madison, WI, USA) by mixing 50 ng of pGEM-T Easy Vector, 5 μL of 2× ligation buffer and 1 U of T4 DNA ligase in a 10 µL reaction volume. After ligation at room temperature for 1 h, 2 μL of the product and 50 μL of *E. coli* DH5α competent cells were mixed and incubated on ice for 20 min, followed by heat shock at 42 °C for 45 s and cooling on ice for 2 min. Then, 950 μL of Luria–Bertani (LB) broth was added and the cells were incubated at 37 °C for 90 min with shaking at 200 rpm. Subsequently, the culture was plated on LB agar containing 100 ppm ampicillin, 0.5 mM isopropyl β-d-1-thiogalactopyranoside, and 80 μg/mL X-Gal for 12 h at 37 °C. The positive colonies identified after blue-white selection were cultured in 5 mL of LB broth at 37 °C for 16 h with shaking at 180 rpm. Following incubation, the culture was centrifuged at 3500 rpm for 10 min and the plasmid DNA was extracted using a QIAprep Spin Miniprep Kit (Qiagen). After removing the supernatant, the cell pellet was suspended in 250 μL of P1 buffer and transferred to a 1.5 mL tube. Next, 250 μL of P2 buffer and 350 μL of N3 buffer were added before centrifugation at 13,000 rpm for 10 min. The supernatant was transferred to a QIAprep spin column that was then centrifuged at 13,000 rpm for 1 min. After discarding the flow-through, 500 μL of PB buffer was added to the column, which was then centrifuged at 13,000 rpm for 1 min. The column was washed with 750 μL of PE buffer and the plasmid DNA was retrieved in 30 μL of nuclease-free water after a final centrifugation of the column at 13,000 rpm for 1 min.

### DNA Sequencing and Primer Design

The plasmid DNA sequence was analyzed by SolGent (SolGent Co., Daejeon, Korea), which verified that the cDNA was inserted into the pGEM-T Easy vector. After analysis of the DNA sequence, cDNA consensus was identified using the basic local alignment search tool (http://blast.ncbi.nlm.nih.gov/Blast.cgi). The EST-SSR markers were designed using the microsatellite analysis program WebSat and Primer3web version 4.0.0. The SSR sites analyzed were used to prepare the forward and reverse primers. Primer design was based on the following core criteria: (1) primer length ranging from 18 to 27 bp; (2) melting temperature between 55 and 62 °C with 60 °C as the optimum; (3) PCR product size ranging from 150 to 500 bp; and (4) GC% content between 20 and 60%, with an amplification rate larger than 80%.

### PCR amplification and polymorphism analysis

PCR was conducted using a UNO II thermocycler (Biometra GmbH, Göttingen, Germany). As a template, 30 ng of sweet potato genomic DNA was added to 2.5 mM 10× buffer (500 mM KCl; 100 mM Tris–HCl, pH 8.3; 15 mM MgCl2), 20 pmol dNTP mixture, 20 pmol SSR marker primer, and 1 U of Taq DNA polymerase. The initial denaturation was carried out at 96 °C for 5 min, and denaturation was at 96 °C for 1 min. The annealing temperature ranged from 54.5 to 61.5 °C for 30 s, and extension was at 72 °C for 1 min. The final extension temperature was 72 °C for 5 min. After the PCR was complete, 5 µL of the product was loaded onto a QIAxcel capillary gel electrophoresis system (Qiagen) for analysis.

## Results

### Construction of cDNA library and treatment by restriction enzymes

Total RNA was isolated from sweet potato tissue for design of the cDNA library. Electrophoresis analysis showed that the cDNA library amplicon size range was varied. A total of 789 cDNAs were cloned in *E. coli* by employing the pGEM-T Easy vector. To confirm the ligation of the plasmid and the cDNA, the extracted plasmid from *E. coli* was cut with a restriction enzyme (*Eco*RI) and analyzed by 1.0% agarose gel electrophoresis.

### Frequency and distribution of EST-SSR markers in sweet potato

**Fig. 1:**
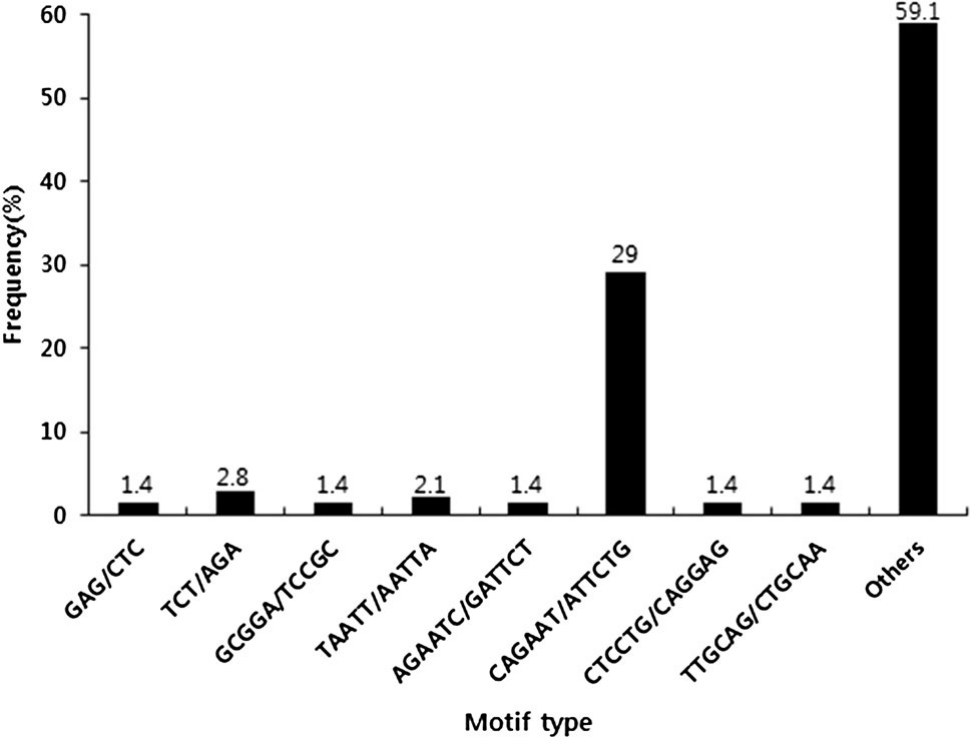
Frequency distribution of EST-derived SSRs of sweet potato based on types of motif sequence. *X* axis is motif sequence types, and *Y* axis represents the frequency of SSRs of a given motif sequence type

A total of 789 ESTs with an average length of 283 bp were used to evaluate the presence of SSR motifs. The length of contigs ranged from 100 to 499 bp, with those >250 bp accounting for 75.7%. A total of 617 SSRs were identified from 579 unique ESTs. Of these, 31 ESTs contained more than one SSR. All the SSRs showed that the ratio of their unit sizes was not evenly distributed. Among the 617 SSRs, the hexanucleotide repeat motifs were the most abundant (512, 82.98%), followed by penta- (69, 11.18%), tri- (28, 4.53%), di- (6, 0.97%), and tetranucleotide (2, 0.32%) repeat motifs (Table 2). As shown in Table 3, the 10–12-bp-long SSRs accounted for 95.5% of the total SSRs, with the remaining sequences being of 14–20 bp in length (28 SSRs, 4.5%). The maximum length of the dinucleotide repeat (TA/TA) and pentanucleotide repeat (GCGAG/CTCGC) was 20 bp, respectively. In addition, a total of 94 SSR motifs were identified of which 4, 4, 2, 56, and 28 were di-, tri-, tetra-, penta-, and hexanucleotide repeats, respectively. The CAGAAT/ATTCTG hexanucleotide repeat was the most abundant motif detected in our designed primer (42, 29.0%), followed by the motifs TCT/AGA (4, 2.8%), TAATT/AATTA (3, 2.1%), GAG/CTC (2, 1.4%), GCGGA/TCCGC (2, 1.4%), AGAATC/GATTCT (2, 1.4%), CTCCTG/CAGGAG (2, 1.4%), and TTGCAG/CTGCAA (2, 1.4%). The frequency of the remaining 84 types of motifs accounted for 59.1% (Fig. 1).

**Table 2:** Summarization of EST-SSR search results

Searched items	Number
Total number of sequences examined	789
Total size of examined sequences (bp)	174,755
Total number of identified SSRs	617
Number of SSR-containing sequences	579
Number of sequences containing more than 1 SSR	31
Dinucleotide	6
Trinucleotide	28
Tetranucleotide	2
Pentanucleotide	69
Hexanucleotide	512

**Table 3:** EST-SSRs based on the number of repeat units for length distribution

Repeat number	Di-	Tri-	Tetra-	Penta-	Hexa-	Total
2	0	0	0	68	512	580
3	0	0	2	0	0	2
4	0	4	0	1	0	5
5	3	24	0	0	0	27
6	0	0	0	0	0	0
7	2	0	0	0	0	2
8	0	0	0	0	0	0
9	0	0	0	0	0	0
10	1	0	0	0	0	1

### Primer design and evaluation of EST-SSR markers in cultivated sweet potato

**Fig. 2:**
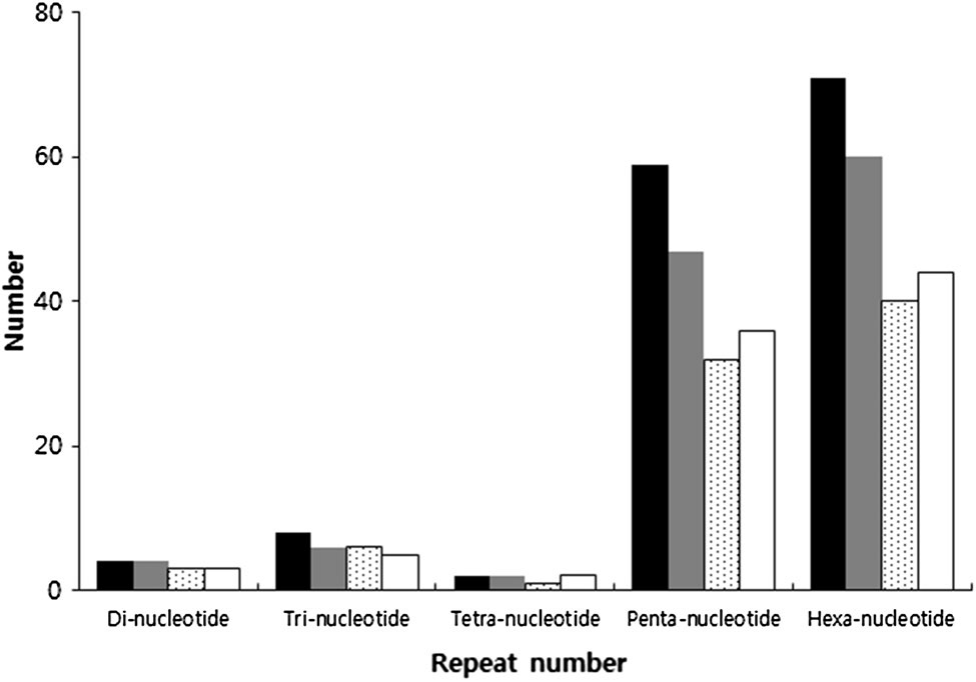
Polymorphic primer pairs and number of designed primer pairs. The figure was indicated number of primer pairs designed (*black columns*), primer pairs amplified (*gray columns*), polymorphic loci in two parents Yeseumi and Annobeny (*dotted white columns*), and polymorphic loci in the 20 sweet potato cultivars (*white columns*)

To ensure the uniqueness of the newly designed EST-SSR primer pairs, 579 potential unique SSR-containing sequences were analyzed. Based on these SSR-containing sequences, 619 pairs of high-quality SSR primers were designed using WebSat (http://www.wsmartins.net/websat/) and Primer3web version 4.0.0 (http://bioinfo.ut.ee/primer3/). The total number of designed primers was 144. Of these, 4, 8, 2, 59, and 71 were for di-, tri-, tetra-, penta- and hexanucleotide repeats, respectively (Fig. 2). After being tested in 20 Korean sweet potato cultivars, 119 primer pairs (82.6%) were successfully amplified. Of these 119 working primer pairs, only 76 amplified PCR products were of the expected sizes. Twenty-four of the other forty-three primers produced larger than expected PCR products and nineteen gave smaller than expected results. The 144 primers were used for further validation in the Yeseumi and Annobeny cultivars, and 106 primer pairs (73.6%) were successfully amplified. Examples of the PCR products amplified in Yeseumi and Annobeny, and in the 20 cultivars, are shown in Fig. 2.

### Polymorphism of EST-SSR markers in cultivated sweet potato

**Fig. 3:**
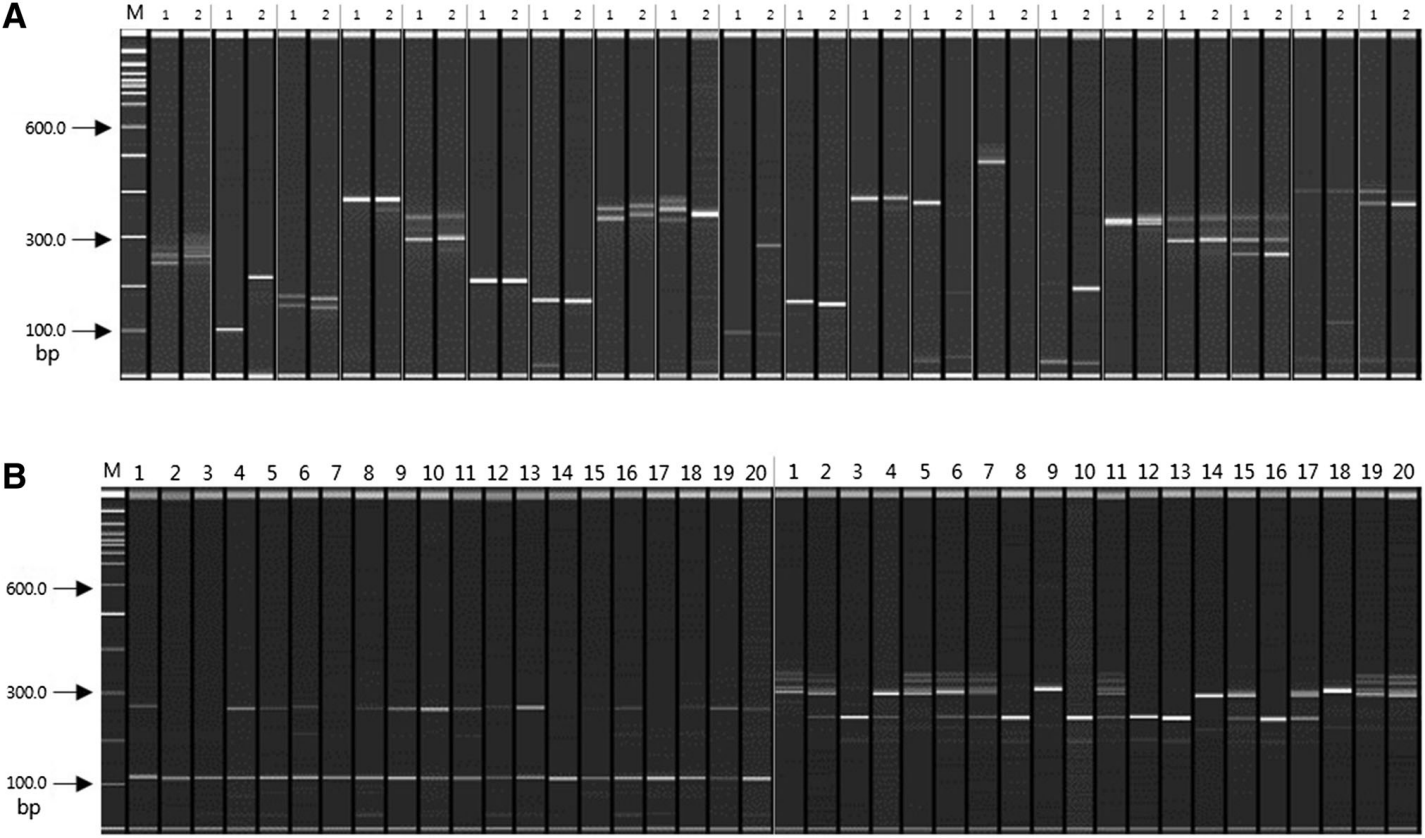
SSR primer pairs for amplification of PCR products. A: PCR products amplified by 20 primer pairs from Yeseumi (*lanes 1*) and Annobeny (*lanes 2*). B: PCR products amplified by 2 primer pairs from twenty sweet potato cultivars. DNA samples from left to right are Yulmi (*lanes 1*), Jeonmi (*lanes 2*), Gogeonmi (*lanes 3*), Jungmi (*lanes 4*), Sincheonmi (*lanes 5*), Geonhwangmi (*lanes 6*), Yeonmi (*lanes 7*), Geonmi (*lanes 8*), Yeonjami (*lanes 9*), Sinjami (*lanes 10*), Sinyulmi (*lanes 11*), Helseumi (*lanes 12*), Geonpungmi (*lanes 13*), Yeseumi (*lanes 14*), Hayanmi (lanes 15), Jinhongmi (*lanes 16*), Juhwangmi (*lanes 17*), Dahomi (*lanes 18*), Simgeonmi (*lanes 19*), and Yeonhwangmi (*lanes 20*). Standard size markers are given on left side

The polymorphism assessment was examined in 20 Korean sweet potato cultivars, as well as Yeseumi and Annobeny. Among the 119 effective SSR primer pairs amplified from the 20 cultivars, 82 (68.9%) were polymorphic (observed for 3 di-, 6 tri-, 1 tetra-, 32 penta-, and 40 hexanucleotide repeats). Among the 106 effective SSR primer pairs amplified from cultivars Yeseumi and Annobeny, 90 (84.9%) were polymorphic (observed for 3 di-, 5 tri-, 2 tetra-, 36 penta-, and 44 hexanucleotide repeats) (Fig. 3).

## Discussion

In this study, we developed EST-SSR markers from the genome sequence of sweet potato. In general, molecular analysis in sweet potato is carried out using SSR markers and inter-simple sequence repeat markers (Hu et al. 2003; Koussao et al. 2014). However, SSR markers do not represent gene function. Recently, the development of an EST-SSR marker in sweet potato was reported, using a next-generation sequencing (Wang et al. 2010) method; namely, isolation of microsatellite markers from genomic sequences. Compared with the traditional methods, such as construction of an SSR-enriched DNA library followed by cloning and sequencing using the Sanger method, next-generation sequencing has the advantages of time and cost savings (Abdelkrim et al. 2009; Santana et al. 2009; Malausa et al. 2011; Zalapa et al. 2012). Nevertheless, although studies using next-generation sequencing have resulted in more than 59 million sequencing reads, it was reported that only 92 primer pairs were successfully employed (Wang et al. 2010). In our study, a total of 789 potential unique EST sequences (about 174.7 kb) were used for searching SSRs, where 579 ESTs (73.4%) contained SSR motifs, generating 617 unique SSRs. Among the 144 designed primers, 119 (82.6%) were successfully amplified in sweet potato, which is a high efficiency rate. In this study, the hexanucleotide repeats were the most frequent motif type, followed by penta-, tri-, di-, and tetranucleotides (Table 2). However, in another report that used a total of 87,492 potential unique EST sequences (about 58.7 Mb) to search for SSRs in sweet potato; of which, 7163 ESTs (8.2%) contained SSR motifs, generating 8294 unique SSRs, the trinucleotide repeat was found to be the most abundant, followed by di-, tetra-, penta-, and hexanucleotides (Wang et al. 2011). In our study, 82 EST-SSR markers among the 20 sweet potato cultivars were polymorphic, whereas 90 EST-SSR markers were polymorphic among the Yeseumi and Annobeny cultivars. We had developed fixed EST-SSR markers in the absence of DNA marker data at sweet potato. This would necessitate a comparison of our fixed EST-SSR markers in more Korean and other cultivars. The development of EST-SSR markers has gained traction in recent years. Qureshi et al. (2004) developed 84 EST-SSR primers of *Gossypium arboreum* var. indium using 9948 sequence data, and Kim et al. (2010) used 30 EST-SSR markers for the analysis of *Rosa hybrida* L. Oh et al. (2010) reported the phylogenetic analysis of *Daucus carota* var. sativa as well as the analysis of various characteristic molecular markers, using 50 markers based on EST sequences. Moreover, besides acting as a molecular tag for particular traits, EST-SSR markers can also simultaneously identify information about the corresponding gene. Thus, by developing EST-SSR markers, the molecular analysis of sweet potato could be done more efficiently. EST-SSR markers can also be used in the construction of sweet potato maps. Thus, we can develop high-quality sweet potato while overcoming the challenges from climate change and other unfavorable conditions.
